# The EU chemicals strategy for sustainability: critical reflections on proposed regulatory changes for endocrine disruptors and mixture toxicity

**DOI:** 10.1007/s00204-022-03227-z

**Published:** 2022-02-05

**Authors:** M. Batke, G. Damm, H. Foth, A. Freyberger, T. Gebel, U. Gundert-Remy, J. Hengstler, A. Mangerich, F. Partosch, C. Röhl, T. Schupp, K. M. Wollin

**Affiliations:** 1 University of Applied Sciences, Emden, Germany; 2 Department of Hepatobiliary Surgery and Visceral Transplantation, University Hospital, Leipzig University, Leipzig, Germany; 3 Institute of Environmental Toxicology, University of Halle, Halle/Saale, Germany; 4 Research and Development, Translational Sciences-Toxicology, Bayer AG, Wuppertal, Germany; 5 Federal Institute for Occupational Safety and Health, Dortmund, Germany; 6 Institute for Clinical Pharmacology and Toxicology, Charité, Universitätsmedizin Berlin, Berlin, Germany; 7 Leibniz Research Centre for Working Environment and Human Factors (IfADo), University of Dortmund, Dortmund, Germany; 8 Molecular Toxicology, Department of Biology, University of Konstanz, Konstanz, Germany; 9 Fraunhofer Institute for Toxicology and Experimental Medicine ITEM, Hannover, Germany; 10 Department of Environmental Health Protection, Schleswig-Holstein State Agency for Social Services, Kiel, Germany; 11 Chemical Engineering, University of Applied Science Muenster, Steinfurt, Germany; 12 Formerly Public Health Agency of Lower Saxony, Hannover, Germany

Currently, the European Commission is pursuing a new "Chemicals Strategy for Sustainability" (CSS) (COM (2020) 667 final). This strategy is part of the European 'Green Deal'. As scientists, we support the European Chemicals Strategy for Sustainability and therefore welcome the efforts to further increase the protection of human health and the environment from hazardous chemicals and to promote innovations for the development of sustainable chemicals. However, some of the anticipated actions would have far-reaching regulatory consequences of questionable added value and that need to be discussed (also see recent considerations in Herzler et al. 2021 and Barile et al. 2021).

In particular, two issues are critical from the perspective of scientists working in the field of human toxicology. These two issues concern (i) substances with endocrine effects and (ii) the question of how to deal with combination effects of chemicals, i.e., mixture toxicity.

## Substances with endocrine effects

Endocrine active chemicals act on endogenous hormonal (i.e., endocrine) systems either by altering hormone concentrations, or by mimicking or inhibiting the action of hormones in the organism. This can be mediated by a wide range of molecular mechanisms. If marked adverse effects are observed following chemical exposures (e.g., disturbances of the thyroid function with the consequence of cardiac arrhythmias), the respective chemicals are called endocrine disruptors. The term ‘endocrine disruptor’ is defined by the World Health Organisation (WHO) restrictively and is used only for exogenous substances or mixtures that alter function(s) of the endocrine system and consequently cause adverse health effects (WHO IPCS 2002).

The new EU-CSS proposes that the system of classification and labelling of hazardous substances (CLP Regulation) shall be extended to include a separate hazard class for endocrine disruptors. In doing so, this would lead to a distinct hazard class based on a specific category of mechanisms of action in addition to the already existing human health hazard classes based on observed adverse effects. Obviously, the planned additional hazard class is based on the assumption that endocrine disruptors are a particularly hazardous group of substances which need to be regulated with specific stringency. However, attention should be paid to the fact that existing legislation already provides a stringent and comprehensive basis for testing and assessing the health effects of substances, including endocrine disruptors. Chemicals causing adverse effects via an endocrine action requiring classification are currently classified as toxic to specific target organ(s), reproductive toxicants, or carcinogens if respective data are available in the literature or from study reports. This follows the same procedure as with chemicals acting via non-endocrine mechanisms. In the past, classification based on observed adverse effects has shown to be an effective approach to protect human health. The new CSS plans, however, would result in a double classification system not in conformity with the internationally established Globally Harmonised System (GHS). With the proposed extension, the European system of classification and labelling would fail to achieve one of its most important goals, namely, to provide targeted information to those who are exposed to the respective substances, with the aim to protect them from potential harm specifically addressed on the label. Obviously, double classification and labelling for effects and mechanisms of action will not lead to better protection of exposed individuals than classification and labelling as it is already in place. Hence, the planned double classification has no added value at least with respect to human health protection.

## Chemical mixtures

Humans and the environment are simultaneously exposed to a large number of chemicals. Up to now, combination effects of chemicals have been taken into account only under specific circumstances and this issue certainly deserves further consideration. The EU proposal aims to account for combination effects using ‘(a) mixture assessment factor(s)’ (SWD (2020a) 250 final). Such mixture assessment factors could be introduced either (i) by a data and evidence-based scientific approach on a case-by-case basis or (ii) by introducing a “generic mixture assessment factor” (SWD (2020b) 248, SWD (2020a) 250). While approach (i) is built on the currently internationally established system of toxicological risk assessment, the underlying assumption made by approach (ii) is that combined exposures to chemicals should be handled, as if they generally exhibit combination effects (i.e., effect of mixture larger as for each of the single mixture components). Thus, in case of introducing a “generic mixture assessment factor”, either guidance values derived under REACH, such as the 'Derived No-Effect Level' (DNEL) values, or the risk quotients, which are set for individual substances, would be reduced by standard factor(s) because of a potential for co-exposure to other chemicals.

However, the assumption that effects of substances in mixtures add up or act synergistically is only applicable under certain premises (European Commission 2012). A combination effect of substances is possible if they trigger an effect via the same initiating event or intervene at some point within the same or related adverse outcome pathways. Even though in cases of different toxicological mechanisms and adverse outcome pathways, the occurrence of combination effects of chemicals cannot fully be ruled out, it is mechanistically improbable and would occur rather as an exception. Moreover, combination effects are not expected for substances acting in a mechanistically independent manner (i.e., ‘response addition’) in cases when co-exposures occur in doses below the established health-based guidance values (e.g., ADI, TDI, DNEL) for the individual substances (European Commission 2012; Boobis et al. 2011). In cases, where the dose–response data of individual substances with related adverse outcome pathways are available, a combination-specific additional safety factor could be determined in a scientifically targeted approach (e.g., see EFSA 2021).

In our view, the introduction of mixture assessment factors deserves further consideration. We advocate to pursue the introduction of mixture assessment factors in a data and evidence-based approach. However, the introduction of a generic mixture assessment factor to deal with chemical co-exposures would be premature, if such a factor would abandon the proven path of a scientifically based risk assessment of chemicals. In this respect, tiered approaches in pesticide and biocide regulation are in place (e.g., Guidance on Biocidal Products Regulation 2017). Ongoing research projects funded by the EU and its member states aim to expand the state of knowledge in this field, which will improve the knowledge basis for future decisions (e.g., see Drakvic et al. 2020; Panoramix 2021). In our opinion, it would be advisable to await the results of these programs and to intensify efforts in this direction to be able to make scientifically sound decisions, as also proposed, e.g., by EuroMix (2019).

## Conclusions

A toxicological health risk assessment approach has been established and successfully worked worldwide for decades. Some of the potential changes in chemicals legislation, e.g., for endocrine disruptors as proposed by the EU Commission, lead to an even stronger emphasis on the intrinsic hazard properties of substances as a regulatory basis (Doe et al. 2021). This would have potentially far-reaching regulatory consequences, some of which would be superfluous from a health risk perspective and without a sound toxicological basis. The latter also holds true for the introduction of a generic mixture toxicity factor, especially as already available and established tiered approaches and current research open up intelligent and specific assessments. Thus, each of the proposed changes needs to be considered carefully in advance from a scientific point of view.
